# Cotard syndrome in a patient with multiple sclerosis: A case report

**DOI:** 10.1192/j.eurpsy.2021.2117

**Published:** 2021-08-13

**Authors:** S. Vieira, G. Marinho

**Affiliations:** 1 Clínica 6, Centro Hospitalar Psiquiátrico de Lisboa, Lisbon, Portugal; 2 Psychiatry, Centro Hospitalar Psiquiátrico de Lisboa, Lisboa, Portugal

**Keywords:** Cotard Syndrome, Multiple sclerosis, case report

## Abstract

**Introduction:**

“Cotard syndrome” is a rare condition characterized by a constellation of clinical features, including hypochondriac and nihilistic delusions, the most characteristic of which are the ideas that one is dead or that their organs do not exist. It is more commonly associated with psychotic depression and schizophrenia but can also be found in several neurological disorders. In the clinical practice it generally appears as an “incomplete Cotard”, reduced to hypochondriac delusions attributed to the malfunction or occlusion of the organs, usually the digestive tract and abdominal viscera. Consequently it is common for these patients to reject food or medications. In literature it has been divided into three types, according to the clinical symptoms: psychotic depression, Cotard type I, and Cotard type II.

**Objectives:**

Literature review on Cotard Syndrome and its link with Multiple Sclerosis, based on a clinical case.

**Methods:**

Pubmed and Google Scholar search using the keywords Cotard Syndrome, Multiple Sclerosis.

**Results:**

Hereby we present a clinical case of a 53-year-old female patient, with multiple sclerosis, who presented with hypochondriac and nihilistic delusions and refusal of food and medication. The patient was treated with olanzapine with rapid remission of delusional activity.

**Conclusions:**

Multiple sclerosis is an immunemediated chronic disease, affecting predominantly the sensory and motor function. In addition, psychiatric comorbidity is very frequent with up to 50 % lifetime risk of depression. While various neurological disorders have been described in association with Cotard syndrome, its link with multiple sclerosis has been scarcely reported.

**Disclosure:**

No significant relationships.

